# Supplementary material for: Significantly improving the quality of genome assemblies through curation
Source: Gigascience. 2021 Jan 9;10(1):giaa153. doi: 10.1093/gigascience/giaa153 (PMC7794651; doi:10.1093/gigascience/giaa153)
Supplement: giaa153_GIGA-D-20-00240_Revision_1 [file giaa153_giga-d-20-00240_revision_1.pdf]

# GigaScience


## Significantly improving the quality of genome assemblies through curation

--Manuscript Draft--

| | |
|---|---|
| **Manuscript Number:** | GIGA-D-20-00240R1 |
| **Full Title:** | Significantly improving the quality of genome assemblies through curation |
| **Article Type:** | Review |
| **Funding Information:** | Wellcome Trust (WT206194) — Not applicable |
| **Abstract:** | Genome sequence assemblies provide the basis for our understanding of biology. Generating error-free assemblies is therefore the ultimate, but sadly still unachieved goal of a multitude of research projects. Despite the ever-advancing improvements in data generation, assembly algorithms and pipelines, no automated approach has so far reliably generated near error-free genome assemblies for eukaryotes.<br>Whilst working towards improved data sets and fully automated pipelines, assembly evaluation and curation is actively employed to bridge this shortcoming and significantly reduce the number of assembly errors. In addition to this increase in product value, the insights gained from assembly curation are fed back into the automated assembly strategy and contribute to notable improvements in genome assembly quality.<br><br>We describe our tried and tested approach for assembly curation using gEVAL, the genome evaluation browser. We outline the procedures applied to genome curation using gEVAL and also our recommendations for assembly curation in an gEVAL-independent context to facilitate the uptake of genome curation in the wider community. |
| **Corresponding Author:** | Kerstin Howe, Dr. rer. nat.<br>Wellcome Sanger Institute<br>Cambridge, UNITED KINGDOM |
| **Corresponding Author Secondary Information:** | |
| **Corresponding Author's Institution:** | Wellcome Sanger Institute |
| **Corresponding Author's Secondary Institution:** | |
| **First Author:** | Kerstin Howe, Dr. rer. nat. |
| **First Author Secondary Information:** | |
| **Order of Authors:** | Kerstin Howe, Dr. rer. nat. |
| | William Chow |
| | Joanna Collins |
| | Sarah Pelan |
| | Damon-Lee Pointon |
| | Ying Sims, PhD |
| | James Torrance, PhD |
| | Alan Tracey |
| | Jonathan Wood |
| **Order of Authors Secondary Information:** | |
| **Response to Reviewers:** | Dear Dr Zauner, |

We have uploaded the revised manuscript “Significantly improving the quality of genome assemblies through curation” by Howe et al.. Thank you very much for your and the reviewer’s comments which very helpful for us improving the paper. We have added illustrations (new Fig.1 and extended Fig. 2) and adapted the text according to the comments to further illustrate and clarify the content.

We hope that you and the reviewers find the revised manuscript much improved. Please find our responses to the editor’s and reviewers’ comments below.

Best regards,

Kerstin Howe

GIGA-D-20-00240
Significantly improving the quality of genome assemblies through curation
Kerstin Howe, Dr. rer. nat.; William Chow; Joanna Collins; Sarah Pelan; Damon-Lee Pointon; Ying Sims; James Torrance, PhD; Alan Tracey; Jonathan Wood
GigaScience

Dear Dr. Howe,

Your Review Article "Significantly improving the quality of genome assemblies through curation" (GIGA-D-20-00240) has been assessed by our reviewers. Based on these reports, and my own assessment as Editor, I am pleased to inform you that it is potentially acceptable for publication in GigaScience, once you have carried out some revisions suggested by our reviewers.

Their reports are below.

I'd like to highlight one point from the reviewers' reports. In the paper, you write that the gEVAL system is not portable and I agree with reviewer 2's remarks that this needs further explanation.

One of GigaScience's aims is to advance sharing and reproducibility and the "original gEVAL manuscript mentions that it is downloadable for use with any organism", as the reviewer says. Would you be able to explain and document in the manuscript which steps are required to set up the gEVAL infrastructure, if another group would like to do so?

Please include a point-by-point within the 'Response to Reviewers' box in the submission system. Please ensure you describe additional experiments that were carried out and include a detailed rebuttal of any criticisms or requested revisions that you disagreed with. Please also ensure that your revised manuscript conforms to the journal style, which can be found in the Instructions for Authors on the journal homepage. If the data and code has been modified in the revision process please be sure to update the public versions of this too.

The due date for submitting the revised version of your article is 07 Jan 2021.

We look forward to receiving your revised manuscript soon.

Best wishes,

Hans Zauner
GigaScience

-----

Reviewer reports:

Reviewer #1: The authors provide a concise overview of issues that arise during efforts to establish a reference quality genome sequence assembly, especially for organisms with complex genomes. The relevant published literature and software sources are cited. Whilst the authors' own infrastructure for reviewing and correcting genome assemblies is an in-house bespoke system that is not portable they describe the key processes involved in reviewing and assessing genome assemblies. This brief editorial / review provides a useful checklist for groups generating genome assemblies. Whilst the generation of the primary sequence data from which a first pass contig level assembly can be built is readily within the capacity of well-founded and funded research groups, the conversion of the resulting contigs into a high quality chromosome level assembly requires time and skill. This review provides a useful guide to navigating this transition and those who aspire to contribute to the growing resource of high quality reference genomes would be well served by reading this guide.

This guide is largely set in the context of the current widely adopted paradigm of single pseudo-haploid representations of an organism's genome. As some of the errors that the procedures described in this paper seek to address concern the challenges of resolving an individual's different haplotypes some comment on graph based genome approaches to capture rather than 'resolve' such haplotypic differences would be appropriate.

Thanks very much to reviewer #1 for the kind assessment of our manuscript. Our curation recommendations are not restricted to pseudo-haplotype-based assemblies, but are also used for haplotype resolved assemblies, where both haplotypes are curated. Full haplotype resolution has its own challenges and these are described in detail in https://www.biorxiv.org/content/10.1101/2020.05.22.110833v1.abstract as cited in the manuscript.

For graph-based assemblies, the current workflow of e.g. the Human Pangenome Project (https://humanpangenome.org/) we are participating in is based on generation of fully haplotype-resolved assemblies first, which are subsequently used to build a graph. We are therefore applying the curation process as described. We are vigilant regarding future requirement and constantly adapt.

For the current manuscript we adapted the conclusions as follows in order to highlight the general suitability of the curation recommendations and hope this addresses the concern:

“Our experiences in curating partially and fully haplotype-resolved genome assemblies for GRC, VGP and DToL have driven improvements in assembly software (e.g. purge_dups [15], salsa2 [52]), assembly pipelines (VGP, DToL) and assembly assessment tools (e.g. Asset [32,38]). Genome assembly generation is a fast-moving field and we are constantly adapting the curation software and processes to include novel data types and novel ways of generating assemblies whilst being conscious of the need to maximise throughput. This ensures ongoing involvement of assembly curation in high-throughput projects to produce the best possible data for the community to base their research upon. This ensures ongoing involvement of assembly curation in high-throughput projects to produce the best possible data for the community to base their research upon.”

Reviewer #2: The authors provide much welcome guidelines and recommendations for genome assembly curation derived from their experience curating hundreds of assemblies. Their recommendations are clear but the manuscript could benefit from more examples of what misassembly signals look like in different technologies. The authors mention that gEVAL is tied into their local infrastructure and not portable, but the original gEVAL manuscript mentions that it is downloadable for use with any organism. It should be made more clear why gEVAL cannot be used. If gEVAL indeed cannot be used outside of their group, it would be nice to see how similar views could be generated with publicly available tools. Finally, I think that it would be hugely beneficial for readers to have a workflow figure with their recommendations

| | |
|---|---|
| | incorporated from the initial coherence check to final ordering and orientation.<br><br>Many thanks to reviewer #2 for the thoughtful comments and suggestions and the detailed corrections.<br><br>Concerns:<br><br>1) Their recommendations are clear but the manuscript could benefit from more examples of what misassembly signals look like in different technologies.<br><br>We have extended Figure 1 (now Fig. 2) to include misassembly signatures detectable in HiC 2D maps, in addition to the already presented signals from read coverage, BioNano maps and synteny analyses. We hope this widens the information from gEVAL-based misassembly information to useful instructions for assessing HiC maps that can be generated with a variety of publicly accessible methods.<br><br>2) It should be made more clear why gEVAL cannot be used.<br><br>When we published the gEVAL paper in 2013, gEVAL was a database for reference genome assemblies maintained by the Genome Reference Consortium. As such it was publicly accessible, and code and database content were offered for download. Whilst the gEVAL browser is still publicly accessible, and the plugins and data described in the publication can be downloaded, the Ensembl version 93 code gEVAL is built on is not publicly available anymore and this is sadly outside our influence. The 2013 publication pertains to all data provided at https://geval.sanger.ac.uk/.<br><br>gEVAL has moved on over nearly a decade and has evolved from a low throughput vehicle for reference curation to a high throughput, fully automated assembly analyser that takes its strength from being totally integrated into the institute's data infrastructure, allowing immediate data retrieval from multiple sources. It is an essential part of the overall assembling pipeline and not promoted as free-standing software. Detangling this to make it publicly available is not possible without additional workforce that we are not funded for. All gEVAL databases we build for the assemblies we are curating are publicly available at vgp-geval.sanger.ac.uk/index.html. This site and its sister site mentioned above are both accessible from geval.org.uk.<br><br>The current manuscript does NOT focus on gEVAL as a software packet, but rather on the process of assembly curation and its importance for generating high quality assemblies. We describe what we have successfully applied for our purposes whilst fully disclosing the logic around assembly curation and the tools publicly available to design an assembly curation pipeline that fits the requirements of the respective user.<br><br>We have amended the manuscript to hopefully explain this better without taking up too much space and distracting from the core message on curation rather than software:<br><br>"gEVAL is tied into our local infrastructure and as such sadly not portable, yet fully publicly accessible at geval.org.uk."<br><br>"The pipeline that GRIT deploys has much evolved since its first implementation [10], and is now so closely tied into the Wellcome Sanger Institute's internal data structure that it cannot be ported, but is described here as an example of a successful implementation that mixes automated and manual processes and significantly improves genome assemblies in a time and resource sensitive way that allows its use within high-throughput projects. All assembly projects loaded into gEVAL are publicly accessible at geval.org.uk."<br><br>The gEVAL functionality can be largely replicated with any tool that visualises sequence and accepts sequence annotation overlays. In the manuscript, we recommend to use ASSET as it also provides the multi-data analyses that are the core of gEVAL. We have extended the text to make this clearer by adding<br><br>"ASSET evaluates multiple data types in parallel and is therefore an excellent tool to |

Powered by Editorial Manager® and ProduXion Manager® from Aries Systems Corporation

assess and visualise potential misassemblies [32].”

3) Finally, I think that it would be hugely beneficial for readers to have a workflow figure with their recommendations incorporated from the initial coherence check to final ordering and orientation.

Thank you for this excellent suggestion, we completely agree and have provided a workflow (Fig. 1) to summarise our recommendations for assembly curation.

Specific comments:

line 100 - extra period at end of sentence

removed

line 106 - spell out Segmental Duplication Assembler.

done

line 113 - comma after "For polishing"

inserted

line 117 - clarify that they can be assembled independently from the raw reads used for genome assembly.

amended: “They can be assembled independently from the raw reads, e.g. using the mitoVGP pipeline”

line 118-119 - This is confusing, it was just stated above that the organelle genome must be included for polishing and now this says to process it independently.

changed from
“Contigs/scaffolds that represent the organelle genomes should be identified and processed independently of the primary, nuclear assembly.”
to
“Contigs/scaffolds that represent the organelle genomes should be identified and submitted as such to the INSDC archives.“
to specify that the different handling applies to the submission process.

line 208 - typo "gata"

corrected to “data”

line 223 - provide a link to a public code repository with the nextflow pipeline

This pipeline is intricately intertwined with the Sanger infrastructure and it would require additional staff to rewrite it to make it publicly useable. A similar public pipeline already exists, and we have added it to the manuscript:

“Before being loaded into gEVAL, all assemblies are run through a nextflow [39] pipeline that performs contamination detection and separation or removal as described in Table 1, combined with removal of trailing Ns [39]. Brief manual checking of the results prevents the erroneous removal of regions likely derived from horizontal gene transfer. This pipeline was inspired by the contamination checking process conducted by Genbank [40].”

Figure 1: This example is a little confusing. It looks like some of the bionano maps agree with the join and span the drop in pacbio read coverage.

The confusion was likely caused by the lack of annotation on the in silico digest tracks and the BioNano map alignments’ colour scheme. We have extended the feature track annotation to the in silico tracks and added further explanations to the figure legend

| | |
|---|---|
| | (yellow = aligned, beige = not aligned BioNano map). |
| Additional Information: | |
| Question | Response |
| Are you submitting this manuscript to a special series or article collection? | No |
| **Experimental design and statistics**<br><br>Full details of the experimental design and statistical methods used should be given in the Methods section, as detailed in our Minimum Standards Reporting Checklist. Information essential to interpreting the data presented should be made available in the figure legends.<br><br>Have you included all the information requested in your manuscript? | Yes |
| **Resources**<br><br>A description of all resources used, including antibodies, cell lines, animals and software tools, with enough information to allow them to be uniquely identified, should be included in the Methods section. Authors are strongly encouraged to cite Research Resource Identifiers (RRIDs) for antibodies, model organisms and tools, where possible.<br><br>Have you included the information requested as detailed in our Minimum Standards Reporting Checklist? | Yes |
| **Availability of data and materials**<br><br>All datasets and code on which the conclusions of the paper rely must be either included in your submission or deposited in publicly available repositories (where available and ethically appropriate), referencing such data using a unique identifier in the references and in the "Availability of Data and Materials" section of your manuscript. | Yes |

Powered by Editorial Manager® and ProduXion Manager® from Aries Systems Corporation

| | |
|---|---|
| Have you have met the above requirement as detailed in our Minimum Standards Reporting Checklist? | |

Manuscript
Click here to access/download;Manuscript;Howe_et_al_revised_manuscript.



# Significantly improving the quality of genome assemblies through curation

Kerstin Howe[1,2], William Chow[1], Joanna Collins[1], Sarah Pelan[1], Damon-Lee Pointon[1], Ying Sims[1], James Torrance[1], Alan Tracey[1], Jonathan Wood[1]

[1]Wellcome Sanger Institute, Cambridge CB10 1SA, UK

[2]Corresponding author

15th June 2020

Autor details:

| | | |
|---|---|---|
| Kerstin Howe | kerstin@sanger.ac.uk | Orcid ID 0000-0003-2237-513X |
| William Chow | wc2@sanger.ac.uk | Orcid ID 0000-0002-9056-201X |
| Joanna Collins | jcc@sanger.ac.uk | Orcid ID 0000-0001-5782-5028 |
| Sarah Pelan | sb2@sanger.ac.uk | Orcid ID 0000-0001-8729-685X |
| Damon-Lee Pointon | dp24@sanger.ac.uk | Orcid ID 0000-0003-2949-6719 |
| Ying Sims | yy5@sanger.ac.uk | Orcid ID 0000-0003-4765-4872 |
| James Torrance | jt8@sanger.ac.uk | Orcid ID 0000-0002-6117-8190 |
| Alan Tracey | alt@sanger.ac.uk | Orcid ID 0000-0002-4805-9058 |
| Jonathan Wood | jmdw@sanger.ac.uk | Orcid ID 0000-0002-7545-2162 |

## Abstract

Genome sequence assemblies provide the basis for our understanding of biology. Generating error-free assemblies is therefore the ultimate, but sadly still unachieved goal of a multitude of research projects. Despite the ever-advancing improvements in data generation, assembly algorithms and pipelines, no automated approach has so far reliably generated near error-free genome assemblies for eukaryotes.

Whilst working towards improved data sets and fully automated pipelines, assembly evaluation and curation is actively employed to bridge this shortcoming and significantly reduce the number of assembly errors. In addition to this increase in product value, the insights gained from assembly curation are fed back into the automated assembly strategy and contribute to notable improvements in genome assembly quality.

We describe our tried and tested approach for assembly curation using gEVAL, the genome evaluation browser. We outline the procedures applied to genome curation using gEVAL and also our recommendations for assembly curation in an gEVAL-independent context to facilitate the uptake of genome curation in the wider community.



## Keywords

Genome, assembly, curation, gEVAL

## Assembly curation adds significant value

Despite the advances in sequencing and mapping technologies and the ever-increasing number of sophisticated algorithms and pipelines available, generating error-free eukaryotic genome assemblies in a purely automated fashion is currently not possible [1,2]. Assembly software designed to generate continuous sequence from raw reads is confused by heterozygous or repeat-rich regions, introducing erroneous duplications, collapses and misjoins. The same issues recur in subsequent scaffolding processes that aim to turn primary contigs into representations of chromosomal units. The fact that these tools are commonly applied in series rather than in parallel results in the passing of mistakes made from one process on to the next. As a result, even so-called high-quality or "platinum" assemblies can suffer from hundreds to thousands of duplications, collapses, misjoins and missed joins. Because assemblies are often judged simply by their continuity, rather than by their completeness and (structural) correctness, these errors go unnoticed. This impacts research in many ways, making whole regions of the genome impossible to access or misleading researchers who misinterpret assembly artefacts as biological findings [3].

One way to address these shortcomings is in-depth analysis of discordances between the assembly that has been generated and the different data types available for the sequenced individual or species and subsequent resolution of these discordances. This can be performed at the sequence and the structural level. Many automated tools are available that assess sequence quality through read alignment, kmer counting, gene finding and other methods [4–7]. For structural quality assessment, several individual tools can be used, but these tend to analyse a single data type at a time rather than combining insights from analysis of several in parallel [8,9].

We created gEVAL, the genome evaluation browser, to enable a user to visualise and evaluate discordances between an assembly and multiple sets of accompanying data at the same time [10]. gEVAL enables the identification of errors and simultaneously suggests ways to resolve them. Combined with manual assessment of the generated data by experienced curators and a pipeline that enables the curators to record changes and recreate the improved assembly accordingly, gEVAL provides a critical addition to strategies striving to produce assemblies of the highest possible quality.

Below we outline the strategic design, achievements and limitations of the gEVAL approach to assembly curation. gEVAL is tied into our local infrastructure and as such sadly not portable, yet fully publicly accessible at geval.org.uk. We therefore also provide detailed recommendations on how to create similar analyses that do not use gEVAL to promote the core, proven design concepts in gEVAL. This is especially timely in the context of emerging projects that aim to assemble the genomes of very large numbers of species to highest quality possible, including the Vertebrate Genomes Project (VGP), the Darwin Tree of Life Project (DToL, darwintreeoflife.org) and the overarching Earth Biogenome Project (EBP) [1,11].

## Checking for assembly coherence, coverage and contamination

We recommend that every genome assembly is checked for coherence. This includes making sure that only data that belong to the relevant species are used for assembly in the first place. This is best checked before starting the assembly process by aligning all raw datasets with e.g. mash [12] and checking that the data are in fact combinable (i.e. that they are likely to derive from the same underlying distribution of sequence). A major source of remaining technical error in assemblies is the retention of duplicated regions that result from failure to recognise

that two sequences are in fact allelic. These false duplications have wide-ranging negative consequences for subsequent research, for example causing prediction of erroneous gene duplications [1]. False duplications are caused by either incorrect resolution of assembly graphs or failures in detection of haplotypic variation. They can be detected using simple read coverage plots or more sophisticated kmer analyses (for example using KAT, the K-mer Analysis Toolkit [5], KMC, the K-mer counter [13] or Merqury [7]. Kmer approaches also support the estimation of the completeness of the assembly (i.e. whether the assembly contains all the relevant kmers present in the reads) and the ploidy of the genome [14]. False duplications can be removed, ideally after generating the contigs, with tools that recognise partial and complete allele overlap, such as purge_dups [15]. In addition to duplications, assembly quality is also negatively affected by erroneous sequence collapses, mostly located in repetitive regions. Collapses are relatively easy to detect based on increased read coverage, but harder to resolve as they require generation of new sequence. This can be performed through extraction of mapped reads and local reassembly under more stringent conditions, or with more sophisticated methods like the Segmental Duplication Assembler (SDA) [16].

Assemblies are frequently polished after contig generation, using the bulk of data or particular high base accuracy data such as Illumina short reads, to correct remaining errors in the derived consensus sequence. It is however possible to over-polish, such that rare repeat variants are replaced by the most abundant version, or where nuclear insertions of organellar genome fragments (nuclear mitochondrial transfers, NUMTs, and nuclear plastid transfers, NUPTs) are polished to match the organelle sequence. For polishing, the target genome assembly therefore must include the organelle genomes. Organellar genomes are often missing from assemblies because assembly toolkits recognise and exclude them as repeat sequence, or because they yield complex graphs that conflict with nuclear insertions. They

can be assembled independently from the raw reads, e.g. using the mitoVGP pipeline [17]. Contigs/scaffolds that represent the organelle genomes should be identified and submitted as such to the INSDC archives.

A preliminary assembly of data from a target species can inadvertently include synthetic sequence from cloning or sequencing systems, contamination from species handled in the same laboratory or sequencing centre, or contamination from natural cobionts of the target (the gut and skin microbiomes, unsuspected parasites, etc.). Decontamination serves to detect and mask or remove sequence not originating from the target species, and to separate organelle genomes from the primary assembly if not carried out previously. This includes identifying remaining vector and adapter contamination based on known sequence. Contaminating sequence can be detected with dedicated toolkits, such as BlobToolKit [18] or Anvi'o [19] or through individual sequence similarity searches using BLAST or Diamond against suitable databases (Table 1). Our in-house pipelines use automated detection of synthetic, laboratory and natural contaminants, but include manual controls to preserve sequences that may be the product of horizontal gene transfer (described below).

Lastly, trailing Ns should be removed from all contigs and scaffolds.

**Table 1**: Detecting decontamination in assemblies, inspired by the processes carried out by GenBank's genome archive [20].

| ***Detection of*** | ***Software tools*** | ***Detection requirements*** | ***Database*** |
|---|---|---|---|
| vector/adapter sequence | Vecscreen [21] | | UniVec [22] |

| | | | |
|---|---|---|---|
| common contaminants | megaBLAST [23] | e-value <= 1e-4, reporting matches >= 98% sequence identity with match length 50-99 bp, >= 94 % with match length 100-199 bp, or >= 90% with match length > 200 bp | Contamination in eukaryotes [24] |
| organelle genomes | megaBLAST | e-value <= 1e-4, sequence identity >= 90%, match length >= 500 | RefSeq mitochondria [25] and plastid [26] assemblies |
| other species | megaBLAST | e-value <= 1e-4, match score >= 100, sequence identity >= 98%; ignore regions also matching highly conserved rDNAs | Windowmasked [27] RefSeq genomes [28] |

## Improving structural integrity

As most assembly pipelines currently apply different scaffolding steps in series, errors in early steps can propagate through the process. To avoid compounding these errors, one could carry out a thorough curation process after every scaffolding step, but if many scaffolding steps are involved this will be very demanding on time and resources. Our experience has shown structural integrity can be successfully improved after completion of a full, automated assembly process [1,10].

The principle behind identification of assembly errors is simple: align all available (raw and other) data to the produced assembly, check for discordances, and then correct. Several tools that detect scaffolding issues with single data types are available, including scaff10x for 10X Chromium linked reads [29], Access for BioNano maps [8], and HiGlass [30], pretext [31] and Juicebox [9] for Hi-C data. ASSET evaluates multiple data types in parallel and is therefore an excellent tool to assess and visualise potential misassemblies [32]. Read coverage plots

identify errors or problem regions through deviation from expected averages (indicating possibly problematic low-coverage regions, haploid regions, or regions of collapsed repeat) and sites where aligned reads are all clipped at the same site (suggesting that the assembly contains an erroneous join). Aligning the assembly against itself can be used to detect duplications.

Additional data not used in generating an assembly also provides critical information. Comparing the assembly to previous assemblies from the same species or to assemblies from closely-related species can highlight areas of disagreement, and thus areas that deserve closer attention during curation. Transcript evidence, as assembled cDNAs or long single-molecule reads, can be aligned to affirm joins across sequence gaps, identify local mis-assemblies, and to detect false duplications. Protein sequences from the same or related species can serve the same purpose. Centromeres and telomeres can be identified in the assembly through sequence features [33,34]. Long-range structural data (such as karyotypes and FISH mapping) and genetic mapping data (such as genetic map or radiation hybrid mapping data) can provide validation of the large-scale correctness of an assembly, and in particular guide correct association and orientation of chromosomal arms with respect to telomeres and centromeres. Chromosome-wide patterns of repeat proportion and GC content can also be used to affirm completeness of chromosomal units.

Once identified, errors should be corrected. We have found that whole genome sequence editing tools such as gap5 [35], are very useful for this process. It is critical to record the corrections made so that the path from primary assembly to the final completed genome assembly is clear and justified.

## Identifying and naming chromosome-scale scaffolds

The ultimate goal of genome assembly is the production of fully contiguous nucleotide sequences that represent each of the chromosomal units for the species, with an estimate of both overall and local quality, and with known sites that may have issues flagged. Long-range data, such as Hi-C contact maps, can reliably indicate which scaffolds correspond to chromosomal units, and these putative chromosomal assemblies can be reconciled with karyotypic information where available. Fully resolved chromosomal units (where all contigs and scaffolds are ordered and oriented) can be submitted to the the INSDC sequence archives (the International Nucleotide Sequence Database Collaboration (INSDC) partners: GenBank, ENA and DDBJ) as a “chromosome”. Scaffolds and contigs that are demonstrably associated with a chromosomal unit but which cannot be joined because of ambiguous order or orientation must be submitted as “unlocalised” for this chromosome. Scaffolds and contigs that cannot be associated with a chromosome, and which also cannot be established as being separate chromosomes, are deemed “unplaced”.

If a reference assembly for the same species or a karyotype with sequence-based anchors is available, chromosome naming should follow the precedent to ensure compatibility with previously reported results. Identification of sex chromosomes can be based on comparisons to related species or from the location of marker genes. In heterogametic individuals, sex chromosomes will also be easily recognisable by their halved sequence coverage compared to automsomes. If no reference for chromosome naming is established, they should be named by size.

Last but not least, every assembly, together with all relevant raw and metadata, should be submitted to one of the INSDC archives (Genbank, ENA or DDBJ, [36]) to allow discoverability, assure community access and provide stability for future analyses.

Fig. 1 summarises the above recommendations in a suggested workflow for assembly curation activities.

## Assembly curation for high-throughput projects

The above described curation processes suffer from the same shortcoming as the assembly process itself: they are usually applied in series rather than in parallel. The benefits of a multitude of data types and approaches are also difficult to realise. Whilst the identification of many assembly issues can be automated, the actual decision to apply a change is still best made by an experienced curator seemingly slowing the process to an extent that excludes it from any high-throughput project.

The Genome Reference Informatics Team (GRIT) assembly curation pipeline was established to deliver high quality assembly curation for the Genome Reference Consortium (GRC, [37]), the VGP and DToL. The pipeline automates the processes of data gathering and computational analysis for decontamination, validation and correction of assemblies, sourcing all available data from in-house and public resources. The analyses are then presented for manual evaluation by experienced genome curators, who perform the evaluation and log required changes. The corrected assembly ready for submission is generated automatically. Central to this pipeline is gEVAL (geval.org.uk), the genome evaluation browser [10]. gEVAL enables visualisation and evaluation of discordances between an assembly and multiple sets of accompanying data in parallel, enabling the simultaneous identification of errors and ways to

resolve them [38]. The pipeline that GRIT deploys has much evolved since its first implementation [10], and is now so closely tied into the Wellcome Sanger Institute's internal data structure that it cannot be ported, but is described here as an example of a successful implementation that mixes automated and manual processes and significantly improves genome assemblies in a time and resource sensitive way that allows its use within high-throughput projects. All assembly projects loaded into gEVAL are publicly accessible at geval.org.uk.

The GRIT curation process usually starts with assemblies that have been purged of duplicates and most haplotypic segments, scaffolded with long-range data and polished. Before being loaded into gEVAL, all assemblies are run through a nextflow [39] pipeline that performs contamination detection and separation or removal as described in Table 1, combined with removal of trailing Ns [39]. Brief manual checking of the results prevents the erroneous removal of regions likely derived from horizontal gene transfer. This pipeline was inspired by the contamination checking process conducted by Genbank [40].

gEVAL analyses are collated in a database built on an Ensembl framework [41] that has been modified to visualise assembly quality rather than gene and feature annotation. Loading of the analyses into gEVAL and subsequent assembly analyses are pipelined using snakemake and vr-runner [42,43]. Which analyses are run and visualised depend on the availability of data, but typically include the types listed in Table 2. The alignments and placements are visualised in a genome browser as feature tracks and colour-coded to indicate agreement or disagreement with the assembly (Fig. 2). The gEVAL process also generates lists that detail discordances between the assembly and the different data types. The process of analysis and loading into gEVAL requires up to 3 days for a 1 Gb assembly.

**Table 2**: Examples of data types and analyses included in gEVAL and their ability to detect issues and errors.

| ***Data type*** | ***Software*** | ***Supports analysis of*** | | | |
|---|---|---|---|---|---|
| | | ***misjoins*** | ***missed joins*** | ***duplications*** | ***collapses*** |
| long reads | Minimap2 [44], winnowmap [45] | x | x | x | x |
| BioNano | BioNano Solve | x | x | x | x |
| 10X linked reads | Break10x [29] | x | | | |
| cDNAs/gene sets | Blat [46] pblat [47] | x | x | x | |
| self-alignments | Mummer [48] | | | x | |
| other assemblies | Compara [41] | x | x | | |
| centromeres | Repeatmasker [41,49] centromere db [33] | x | x | | |
| telomeres | Find_telomere [34] adapted to work with any sequence | x | x | | |
| genetic and other maps | EPCR [50], Blast [51] | x | x | x | |

gEVAL automatically flags areas where the raw and other comparative data available are discordant with the presented assembly. Experienced curators use the gEVAL database and visualisation, and (where available) Hi-C maps (generated outside the gEVAL pipeline and viewed in HiGlass [30], or pretext [31], to check each listed discordance and decide whether and how to adjust the sequence based on the available data (Fig. 2). In rare cases, the information contained in gEVAL and the Hi-C maps is not sufficient to decide whether a change is warranted. The curators then use additional tools such as gap5 [35] for in-depth

analysis of aligned reads or Genomicus for information on synteny with other species [52]. Curators propose a variety of interventions such as breaking or joining sequence regions, changing the order and orientation of scaffolds and contigs and removing false duplications. Detangling sequence collapses is currently only possible where additional data can be employed for local reassembly. In high-throughput projects such as DToL or VGP curation is usually restricted to a resolution of around 100 kb. This allows an experienced curator to complete curation of 1 Gb of sequence in around 3 days. For projects without immediate time restraints and aimed at single references, such as the genomes curated within the GRC, there is no resolution limit.

During the gEVAL build, assembly scaffolds are split into equally sized components, with their order and orientation recorded in a path file under version control, listing component name, scaffold name and orientation. Should any rearrangement be necessary, the curators simply reorder/reorient the components in the path file. If necessary, components can be split with bespoke scripts which create new components and store them in the gEVAL database. After manual curation, the adjusted ordering and orientation of components and a list of scaffold-chromosome associations is processed automatically to generate the final assembly for submission. All milestones and metrics of the whole curation process are recorded in a tracking database.

## Using gEVAL to assess published assemblies

Above we have described the use of gEVAL to create high-quality assemblies. gEVAL can also also be used to support research communities in verifying research results, ensuring they are not based on assembly artefacts. For this, a gEVAL database is generated for publicly available

assemblies, as e.g. is the case for all GRC assemblies [38]. Here, gEVAL offers the same analyses as detailed above, plus additional databases with other assemblies of the same species, such as previous versions of the current reference, including whole genome alignments between them (Fig. 3). Combined with tutorials and documentation, this provides a valuable resource for users of the featured reference assemblies.

## Impact of assembly curation for high throughput-projects

During curation of 111 assemblies (174 Gb sequence) for VGP and DToL, on average 221 interventions per Gb of sequence were applied (67 breaks, 105 joins and 49 removals of false duplications, Fig. 4). These changes led to an average reduction in assembly length by 2% as the curation effort did not generate new sequence. However, average scaffold N50 increased by 40% and scaffold number decreased by 29%. It is important to note that scaffold N50 changes differed for each assembly, and that while the process improved N50 several hundred fold in initially fragmented assemblies it halved the N50 in over-scaffolded assemblies. On average 96% of assembly sequence was scaffolded to chromosome-level (Fig. 5). The number and scale of changes to the assemblies necessary across the diversity of species analysed shows the persistent need for manual intervention on the path to high quality genome assemblies. Our experiences in curating partially and fully haplotype-resolved genome assemblies for GRC, VGP and DToL have driven improvements in assembly software (e.g. purge_dups [15], salsa2 [53]), assembly pipelines (VGP, DToL) and assembly assessment tools (e.g. Asset [32,38]). Genome assembly generation is a fast-moving field and we are constantly adapting the curation software and processes to include novel data types and novel ways of generating assemblies whilst being conscious of the need to maximise

throughput. This ensures ongoing involvement of assembly curation in high-throughput projects to produce the best possible data for the community to base their research upon.

## Acknowledgements

We thank Mark Blaxter, Richard Durbin and Shane McCarthy for their input on this project and many helpful discussions. Additional thanks go to Mark Blaxter for many constructive discussions around this manuscript. The genome curation project is heavily influenced by the Genome Reference Consortium, the Vertebrate Genomes Project and the Darwin Tree of Life Project and we are indebted to all the members for their engagement with the curation process. This project is supported by the Wellcome Trust, WT206194.

## References

1. Rhie A, McCarthy SA, Fedrigo O, Damas J, Formenti G, Koren S, et al. Towards complete and error-free genome assemblies of all vertebrate species [Internet]. bioRxiv. 2020 [cited 2020 Jul 13]. p. 2020.05.22.110833. Available from: https://www.biorxiv.org/content/10.1101/2020.05.22.110833v1.abstract

2. Miga KH, Koren S, Rhie A, Vollger MR, Gershman A, Bzikadze A, et al. Telomere-to-telomere assembly of a complete human X chromosome. Nature [Internet]. 2020; Available from: http://dx.doi.org/10.1038/s41586-020-2547-7

3. Ko BJ, Lee C, Kim J, Rhie A, Yoo DA, Cho S, Howe K, Wood JMD, VGP assembly group, Jarvis ED and Kim H. Widespread false gene gains caused by duplication errors in genome assemblies. In preparation. 2020;

4. Yang L-A, Chang Y-J, Chen S-H, Lin C-Y, Ho J-M. SQUAT: a Sequencing Quality Assessment Tool for data quality assessments of genome assemblies. BMC Genomics. 2019;19:238.

5. Mapleson D, Garcia Accinelli G, Kettleborough G, Wright J, Clavijo BJ. KAT: a K-mer analysis toolkit to quality control NGS datasets and genome assemblies. Bioinformatics. 2017;33:574–6.

6. Seppey M, Manni M, Zdobnov EM. BUSCO: Assessing Genome Assembly and Annotation Completeness [Internet]. Methods in Molecular Biology. 2019. p. 227–45. Available from: http://dx.doi.org/10.1007/978-1-4939-9173-0_14

7. Rhie A, Walenz BP, Koren S, Phillippy AM. Merqury: reference-free quality, completeness, and phasing assessment for genome assemblies [Internet]. Available from: http://dx.doi.org/10.1101/2020.03.15.992941

8. Chan S, Lam E, Saghbini M, Bocklandt S, Hastie A, Cao H, et al. Structural Variation

Detection and Analysis Using Bionano Optical Mapping. Copy Number Variants. Humana Press, New York, NY; 2018. p. 193–203.

9. Durand NC, Robinson JT, Shamim MS, Machol I, Mesirov JP, Lander ES, et al. Juicebox Provides a Visualization System for Hi-C Contact Maps with Unlimited Zoom. Cell Syst. 2016;3:99–101.

10. Chow W, Brugger K, Caccamo M, Sealy I, Torrance J, Howe K. gEVAL - a web-based browser for evaluating genome assemblies. Bioinformatics. 2016;32:2508–10.

11. Lewin HA, Robinson GE, Kress WJ, Baker WJ, Coddington J, Crandall KA, et al. Earth BioGenome Project: Sequencing life for the future of life. Proc Natl Acad Sci U S A. 2018;115:4325–33.

12. Rhie A. Mash Pipeline [Internet]. [cited 2020 Jul 17]. Available from: https://github.com/VGP/vgp-assembly/tree/master/pipeline/mash

13. van Haarst J Plaza Oñate F Karasikov M KMSSDS. KMC [Internet]. [cited 2020 Jul 17]. Available from: https://github.com/refresh-bio/KMC

14. Rhyker Ranallo-Benavidez T, Jaron KS, Schatz MC. GenomeScope 2.0 and Smudgeplot for reference-free profiling of polyploid genomes. Nat Commun. Nature Publishing Group; 2020;11:1–10.

15. Guan D, McCarthy SA, Wood J, Howe K, Wang Y, Durbin R. Identifying and removing haplotypic duplication in primary genome assemblies. Bioinformatics. 2020;36:2896–8.

16. Vollger MR, Dishuck PC, Sorensen M, Welch AE, Dang V, Dougherty ML, et al. Long-read sequence and assembly of segmental duplications. Nat Methods. 2019;16:88–94.

17. Formenti G, Rhie A, Balacco J, Haase B, Mountcastle J, Fedrigo O, et al. Complete vertebrate mitogenomes reveal widespread gene duplications and repeats [Internet]. bioRxiv. 2020 [cited 2020 Jul 13]. p. 2020.06.30.177956. Available from:

https://www.biorxiv.org/content/10.1101/2020.06.30.177956v1.abstract

18. Challis R, Richards E, Rajan J, Cochrane G, Blaxter M. BlobToolKit - Interactive Quality Assessment of Genome Assemblies. G3 . 2020;10:1361–74.

19. Eren AM, Murat Eren A, Esen ÖC, Quince C, Vineis JH, Morrison HG, et al. Anvi'o: an advanced analysis and visualization platform for 'omics data [Internet]. PeerJ. 2015. p. e1319. Available from: http://dx.doi.org/10.7717/peerj.1319

20. Contamination in sequence databases [Internet]. [cited 2020 Jul 17]. Available from: https://www.ncbi.nlm.nih.gov/tools/vecscreen/contam/

21. Hancock JM, Bishop MJ. VecScreen [Internet]. Dictionary of Bioinformatics and Computational Biology. 2004. Available from: http://dx.doi.org/10.1002/9780471650126.dob0783.pub2

22. UniVec [Internet]. [cited 2020 Jul 17]. Available from: ftp://ftp.ncbi.nlm.nih.gov/pub/UniVec/

23. Morgulis A, Coulouris G, Raytselis Y, Madden TL, Agarwala R, Schäffer AA. Database indexing for production MegaBLAST searches. Bioinformatics. 2008;24:1757–64.

24. Contamination in eukaryotes [Internet]. [cited 2020 Jul 17]. Available from: ftp://ftp.ncbi.nlm.nih.gov/pub/kitts/contam_in_euks.fa.gz

25. RefSeq. RefSeq assemblies: mitochondria [Internet]. [cited 2020 Jul 17]. Available from: ftp://ftp.ncbi.nlm.nih.gov/refseq/release/mitochondrion/

26. RefSeq. RefSeq assemblies: plastids [Internet]. [cited 2020 Jul 17]. Available from: ftp://ftp.ncbi.nlm.nih.gov/refseq/release/plastid/

27. Morgulis A, Gertz EM, Schäffer AA, Agarwala R. WindowMasker: window-based masker for sequenced genomes. Bioinformatics. 2006;22:134–41.

28. O'Leary NA, Wright MW, Brister JR, Ciufo S, Haddad D, McVeigh R, et al. Reference

sequence (RefSeq) database at NCBI: current status, taxonomic expansion, and functional annotation. Nucleic Acids Res. 2016;44:D733–45.

29. Ning Z HE. Scaff10X v4.2: Pipeline for scaffolding and breaking a genome assembly using 10x genomics linked-reads [Internet]. [cited 2020 Jul 17]. Available from: https://github.com/wtsi-hpag/Scaff10X

30. Kerpedjiev P, Abdennur N, Lekschas F, McCallum C, Dinkla K, Strobelt H, et al. HiGlass: web-based visual exploration and analysis of genome interaction maps. Genome Biol. 2018;19:125.

31. Harry E. PretextView (Paired REad TEXTure Viewer): A desktop application for viewing pretext contact maps [Internet]. [cited 2020 Jul 17]. Available from: https://github.com/wtsi-hpag/PretextView

32. Guan D. Asset: An assembly evaluation tool [Internet]. [cited 2020 Jul 17]. Available from: https://github.com/dfguan/asset

33. Melters DP, Bradnam KR, Young HA, Telis N, May MR, Ruby JG, et al. Comparative analysis of tandem repeats from hundreds of species reveals unique insights into centromere evolution. Genome Biol. 2013;14:R10.

34. Koren S. Find_telomere [Internet]. [cited 2020 Jul 17]. Available from: https://github.com/VGP/vgp-assembly/tree/master/pipeline/telomere

35. Bonfield JK, Whitwham A. Gap5--editing the billion fragment sequence assembly. Bioinformatics. 2010;26:1699–703.

36. Karsch-Mizrachi I, Takagi T, Cochrane G, International Nucleotide Sequence Database Collaboration. The international nucleotide sequence database collaboration. Nucleic Acids Res. 2018;46:D48–51.

37. Church DM, Schneider VA, Graves T, Auger K, Cunningham F, Bouk N, et al. Modernizing

reference genome assemblies. PLoS Biol. 2011;9:e1001091.

38. Genome Reference Informatics Team. gEVAL: The Genome Evaluation Browser [Internet]. [cited 2020 Jul 17]. Available from: https://geval.org.uk/

39. Di Tommaso P, Chatzou M, Floden EW, Barja PP, Palumbo E, Notredame C. Nextflow enables reproducible computational workflows. Nat Biotechnol. 2017;35:316–9.

40. ContamFilter [Internet]. Github; [cited 2020 Nov 12]. Available from: https://github.com/NCBI-Hackathons/ContamFilter

41. Aken BL, Achuthan P, Akanni W, Amode MR, Bernsdorff F, Bhai J, et al. Ensembl 2017. Nucleic Acids Res. 2017;45:D635–42.

42. Köster J, Rahmann S. Snakemake-a scalable bioinformatics workflow engine. Bioinformatics. 2018;34:3600.

43. Danecek P, McCarthy S, Randall JC, Bala S, Noell G. vr-runner: A lightweight pipeline framework [Internet]. [cited 2020 Jul 17]. Available from: https://github.com/VertebrateResequencing/vr-runner

44. Li H. Minimap and miniasm: fast mapping and de novo assembly for noisy long sequences. Bioinformatics. 2016;32:2103–10.

45. Jain C, Rhie A, Zhang H, Chu C, Koren S, Phillippy A. Weighted minimizer sampling improves long read mapping [Internet]. Available from: http://dx.doi.org/10.1101/2020.02.11.943241

46. Kent WJ. BLAT---The BLAST-Like Alignment Tool [Internet]. Genome Research. 2002. p. 656–64. Available from: http://dx.doi.org/10.1101/gr.229202.

47. Wang M, Kong L. Pblat: A Multithread Blat Algorithm Speeding Up Aligning Sequences to Genomes. BMC Bioinformatics [Internet]. BMC Bioinformatics; 2019 [cited 2020 Jul 13];20. Available from: https://pubmed.ncbi.nlm.nih.gov/30646844/

48. Kurtz S, Phillippy A, Delcher AL, Smoot M, Shumway M, Antonescu C, et al. Versatile and open software for comparing large genomes. Genome Biol. 2004;5:R12.

49. Bao W, Kojima KK, Kohany O. Repbase Update, a database of repetitive elements in eukaryotic genomes. Mob DNA. 2015;6:11.

50. Shyu C, Foster JA, Forney LJ. Electronic polymerase chain reaction (EPCR) search algorithm [Internet]. Proceedings. IEEE Computer Society Bioinformatics Conference. Available from: http://dx.doi.org/10.1109/csb.2002.1039361

51. Altschul SF, Gish W, Miller W, Myers EW, Lipman DJ. Basic local alignment search tool. J Mol Biol. 1990;215:403–10.

52. Nguyen NTT, Vincens P, Roest Crollius H, Louis A. Genomicus 2018: karyotype evolutionary trees and on-the-fly synteny computing. Nucleic Acids Res. 2018;46:D816–22.

53. Ghurye J, Pop M, Koren S, Bickhart D, Chin C-S. Scaffolding of long read assemblies using long range contact information. BMC Genomics. 2017;18:527.

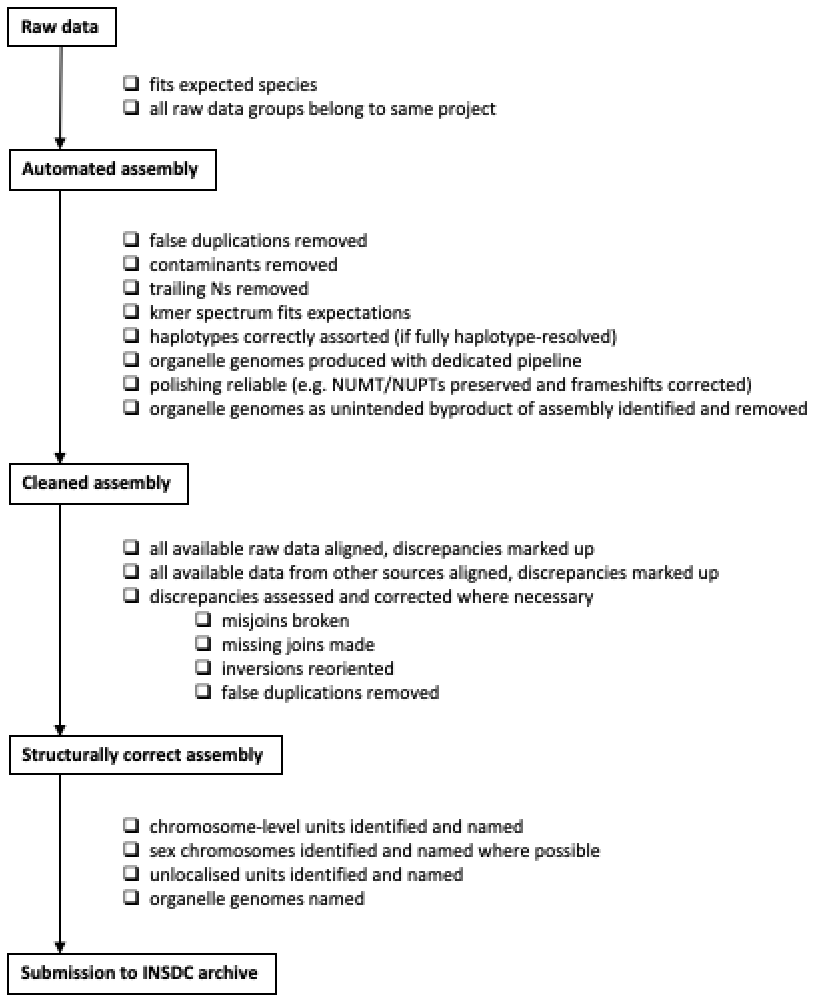


**Figure 1**: Recommended workflow for curation activities during assembly generation.

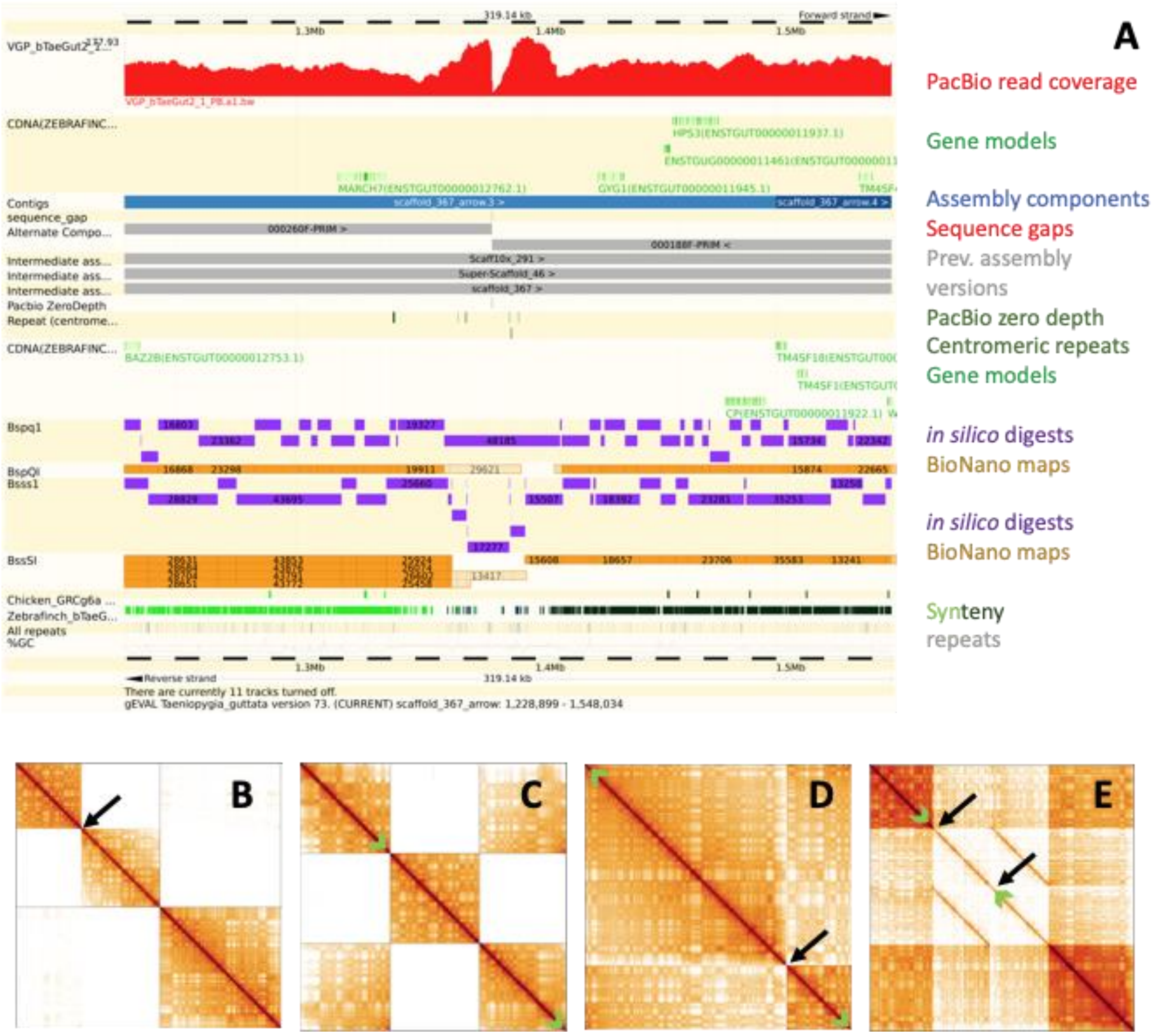


**Figure 2**: Examples of assembly error signatures in different data types. **(A)** Assembly issue identified in gEVAL in a bird genome (*Taeniopygia guttata*, VGP). Feature tracks (named on the right) are shown in the context of the assembly. A misjoin is visible in the middle of the example, indicated by the drop in Pacific Biosciences read coverage, discordance with the aligned (yellow = aligned, beige = not aligned) BioNano maps and the break in synteny. The alignments with intermediate assembly stages show that this error was introduced by the scaffolding step involving scaff10x. **(B-E)** Assembly issues identified in HiGlass HiC 2D maps of a human assembly (HG002, varying assembly approaches). Scaffold boundaries are delineated in gray. **(B)** The first of the two scaffolds depicted here shows a misjoin (black arrow) that needs to be broken. The second scaffold reveals no structural issues. **(C)** The first and third of

the three scaffolds shown here need to be joined as indicated by the green arrows. **(D)** The single scaffold depicted here has a misjoin (black arrow) that needs to be broken and rejoined as indicated by the green arrows. **(E)** This single scaffold contains a duplication, half of which needs to be excised (e.g. black arrows) and the scaffold rejoined (green arrows). The choice of the excised half can be based on phasing.

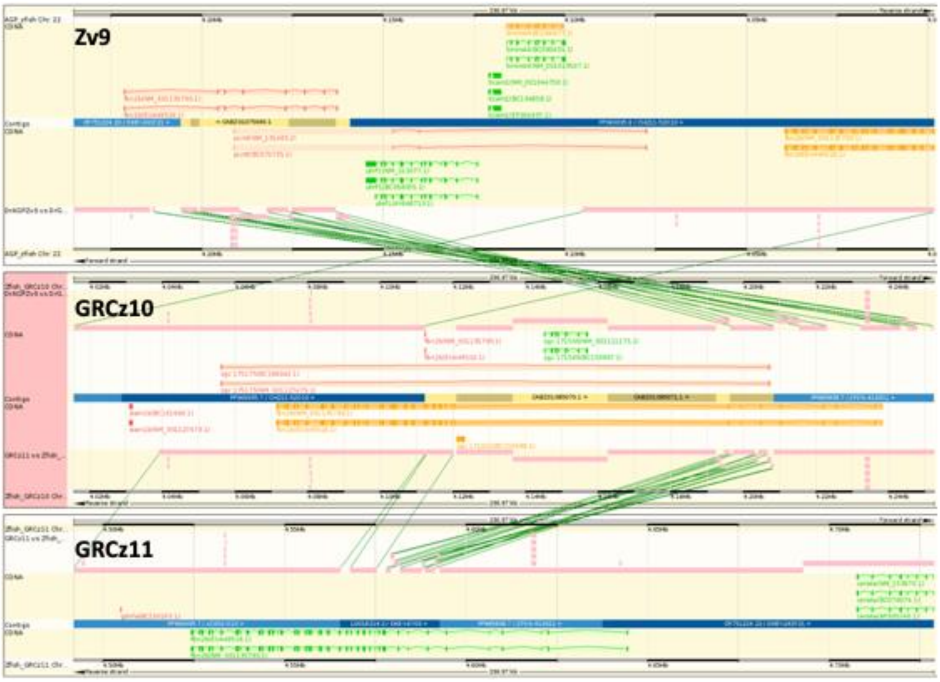


**Figure 3:** Comparison of the *fbn2b* region in the *Danio rerio* (zebrafish) reference assemblies Zv9 (top), GRCz10 (middle) and GRCz11 (bottom) in gEVAL. The fragmented *fbn2b* locus (colour coded in orange and red) was adjusted for GRCz10 (colour coded in orange) and further improved by removing whole genome shotgun contigs in favour of finished clone sequence for GRCz11. The final correct gene locus is coloured in green.

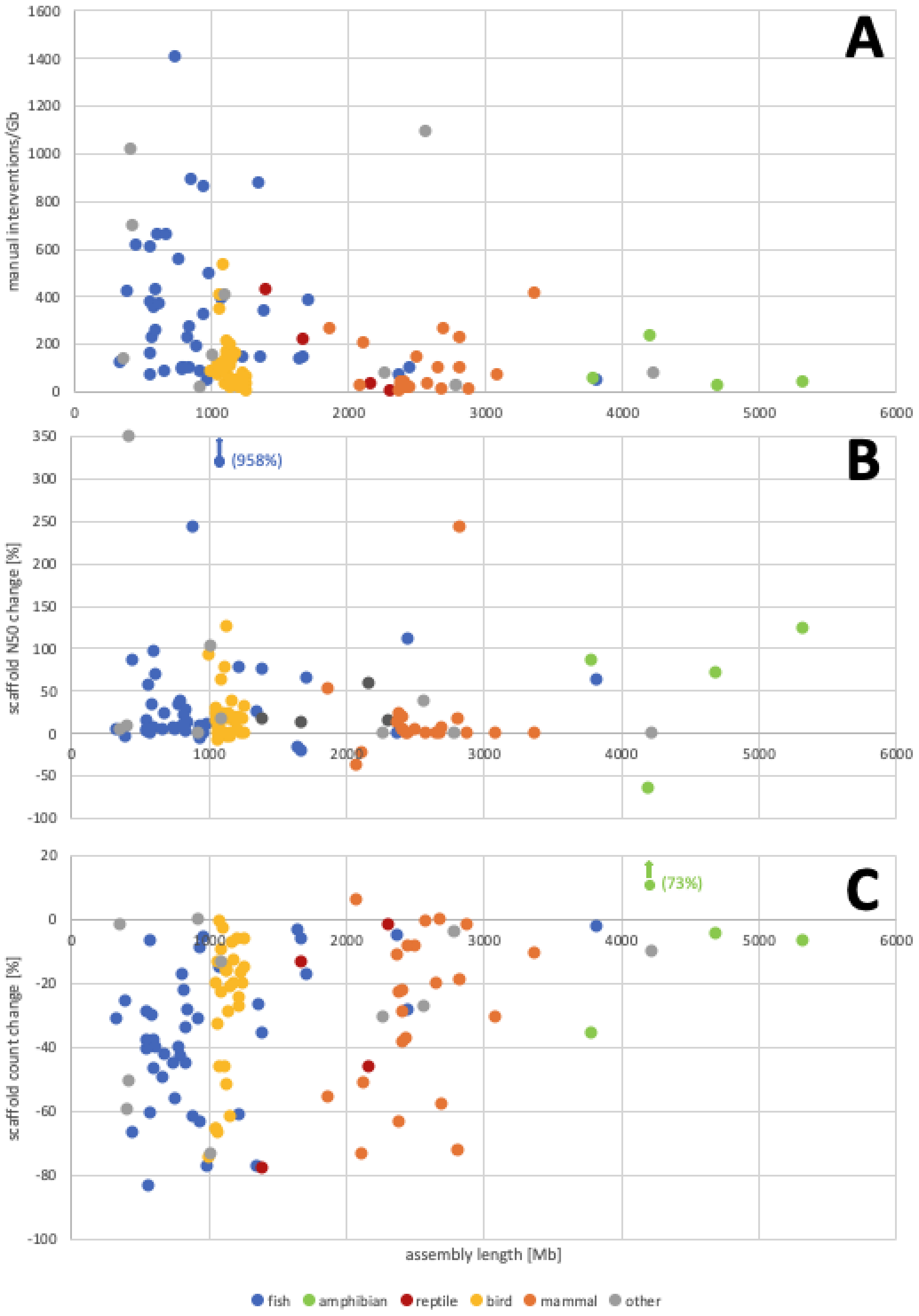


**Figure 4**: Changes to 111 assemblies from different clades through manual assembly curation by the Genome Reference Informatics Team at the Wellcome Sanger Institute. (**A**) Manual interventions (breaks, joins, removal of false duplications) as events per Gb of

assembly sequence. (**B**) Changes in scaffold N50 after curation. (**C**) Changes in scaffold counts after curation. The depicted assemblies were created with PacBio CLR, Chromium 10X, BioNano and Hi-C data.

**A** **B**

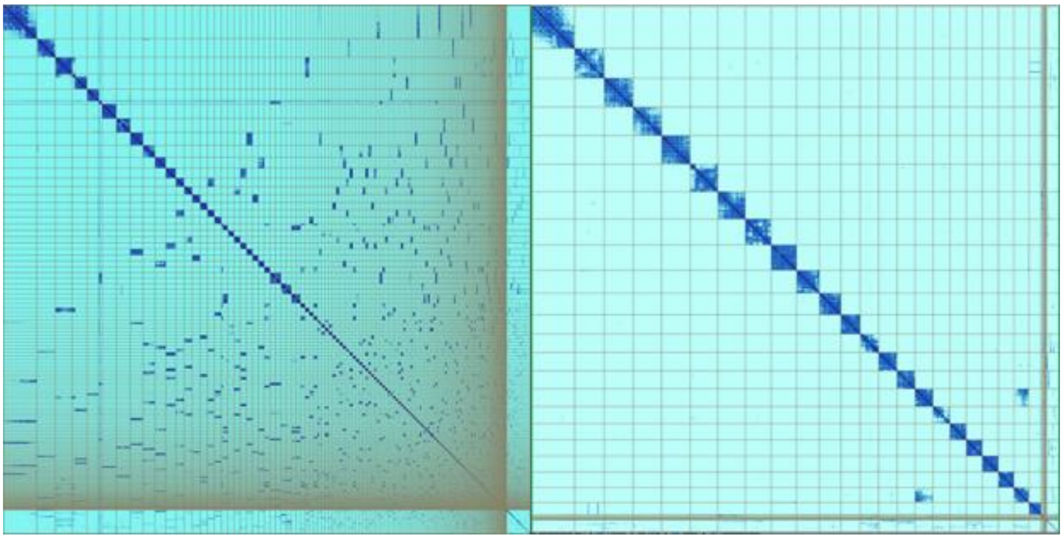

**Figure 5**: Hi-C maps (pretext) showing the *Asterias rubens* (starfish) genome assembly (sequenced as part of the Sanger Institute's 25 genomes for 25 years project) before (**A**) and after (**B**) curation. The curation corrected the initial assembly by making 75 breaks and 216 joins and removed one stretch of erroneously duplicated sequence. 97% of the assembly sequence could be assigned to 22 chromosomes. The curated assembly (**B**) contains one scaffold that is known to be associated with a second one (off-diagonal signal at bottom right), but its order and orientation are ambiguous. This scaffold has been submitted as "unlocalised" for the relevant chromosome.
