# Supplementary material for: Significantly improving the quality of genome assemblies through curation
Source: Gigascience. 2021 Jan 9;10(1):giaa153. doi: 10.1093/gigascience/giaa153 (PMC7794651; doi:10.1093/gigascience/giaa153)
Supplement: giaa153_Response_to_Reviewer_Comments_Original_Submission [file giaa153_response_to_reviewer_comments_original_submission.pdf]

Author's Response To Reviewer Comments

Close

Dear Dr Zauner,

We have uploaded the revised manuscript “Significantly improving the quality of genome assemblies through curation” by Howe et al.. Thank you very much for your and the reviewer’s comments which very helpful for us improving the paper. We have added illustrations (new Fig.1 and extended Fig. 2) and adapted the text according to the comments to further illustrate and clarify the content.

We hope that you and the reviewers find the revised manuscript much improved. Please find our responses to the editor’s and reviewers’ comments below.

Best regards,

Kerstin Howe

GIGA-D-20-00240
Significantly improving the quality of genome assemblies through curation
Kerstin Howe, Dr. rer. nat.; William Chow; Joanna Collins; Sarah Pelan; Damon-Lee Pointon; Ying Sims; James Torrance, PhD; Alan Tracey; Jonathan Wood
GigaScience

Dear Dr. Howe,

Your Review Article "Significantly improving the quality of genome assemblies through curation" (GIGA-D-20-00240) has been assessed by our reviewers. Based on these reports, and my own assessment as Editor, I am pleased to inform you that it is potentially acceptable for publication in GigaScience, once you have carried out some revisions suggested by our reviewers.

Their reports are below.

I'd like to highlight one point from the reviewers' reports. In the paper, you write that the gEVAL system is not portable and I agree with reviewer 2's remarks that this needs further explanation.

One of GigaScience's aims is to advance sharing and reproducibility and the "original gEVAL manuscript mentions that it is downloadable for use with any organism", as the reviewer says. Would you be able to explain and document in the manuscript which steps are required to set up the gEVAL infrastructure, if another group would like to do so?

Please include a point-by-point within the 'Response to Reviewers' box in the submission system. Please ensure you describe additional experiments that were carried out and include a detailed rebuttal of any criticisms or requested revisions that you disagreed with. Please also ensure that your revised manuscript conforms to the journal style, which can be found in the Instructions for Authors on the journal homepage. If the data and code has been modified in the revision process please be sure to update the public versions of this too.

The due date for submitting the revised version of your article is 07 Jan 2021.

We look forward to receiving your revised manuscript soon.

Best wishes,

Hans Zauner
GigaScience

Reviewer reports:

Reviewer #1: The authors provide a concise overview of issues that arise during efforts to establish a reference quality genome sequence assembly, especially for organisms with complex genomes. The relevant published literature and software sources are cited. Whilst the authors' own infrastructure for reviewing and correcting genome assemblies is an in-house bespoke system that is not portable they describe the key processes involved in reviewing and assessing genome assemblies. This brief editorial / review provides a useful checklist for groups generating genome assemblies. Whilst the generation of the primary sequence data from which a first pass contig level assembly can be built is readily within the capacity of well-founded and funded research groups, the conversion of the resulting contigs into a high quality chromosome level assembly requires time and skill. This review provides a useful guide to navigating this transition and those who aspire to contribute to the
growing resource of high quality reference genomes would be well served by reading this guide.
This guide is largely set in the context of the current widely adopted paradigm of single pseudo-haploid representations of an organism's genome. As some of the errors that the procedures described in this paper seek to address concern the challenges of resolving an individual's different haplotypes some comment on graph based genome approaches to capture rather than 'resolve' such haplotypic differences would be appropriate.

Thanks very much to reviewer #1 for the kind assessment of our manuscript. Our curation recommendations are not restricted to pseudo-haplotype-based assemblies, but are also used for haplotype resolved assemblies, where both haplotypes are curated. Full haplotype resolution has its own challenges and these are described in detail in
https://www.biorxiv.org/content/10.1101/2020.05.22.110833v1.abstract as cited in the manuscript.

For graph-based assemblies, the current workflow of e.g. the Human Pangenome Project (https://humanpangenome.org/) we are participating in is based on generation of fully haplotype-resolved assemblies first, which are subsequently used to build a graph. We are therefore applying the curation process as described. We are vigilant regarding future requirement and constantly adapt.

For the current manuscript we adapted the conclusions as follows in order to highlight the general suitability of the curation recommendations and hope this addresses the concern:

"Our experiences in curating partially and fully haplotype-resolved genome assemblies for GRC, VGP and DToL have driven improvements in assembly software (e.g. purge_dups [15], salsa2 [52]), assembly pipelines (VGP, DToL) and assembly assessment tools (e.g. Asset [32,38]). Genome assembly generation is a fast-moving field and we are constantly adapting the curation software and processes to include novel data types and novel ways of generating assemblies whilst being conscious of the need to maximise throughput. This ensures ongoing involvement of assembly curation in high-throughput projects to produce the best possible data for the community to base their research upon. This ensures ongoing involvement of assembly curation in high-throughput projects to produce the best possible data for the community to base their research upon."

Reviewer #2: The authors provide much welcome guidelines and recommendations for genome assembly curation derived from their experience curating hundreds of assemblies. Their recommendations are clear but the manuscript could benefit from more examples of what misassembly signals look like in different technologies. The authors mention that gEVAL is tied into their local infrastructure and not portable, but the original gEVAL manuscript mentions that it is downloadable for use with any organism. It should be made more clear why gEVAL cannot be used. If gEVAL indeed cannot be used outside of their group, it would be nice to see how similar views could be generated with publicly available tools. Finally, I think that it would be hugely beneficial for readers to have a workflow figure with their recommendations incorporated from the initial coherence check to final ordering and orientation.

Many thanks to reviewer #2 for the thoughtful comments and suggestions and the detailed corrections.

Concerns:

1) Their recommendations are clear but the manuscript could benefit from more examples of what misassembly signals look like in different technologies.

We have extended Figure 1 (now Fig. 2) to include misassembly signatures detectable in HiC 2D maps, in addition to the already presented signals from read coverage, BioNano maps and synteny analyses. We hope this widens the information from gEVAL-based misassembly information to useful instructions for assessing HiC maps that can be generated with a variety of publicly accessible methods.

2) It should be made more clear why gEVAL cannot be used.

When we published the gEVAL paper in 2013, gEVAL was a database for reference genome assemblies maintained by the Genome Reference Consortium. As such it was publicly accessible, and code and database content were offered for download. Whilst the gEVAL browser is still publicly accessible, and the plugins and data described in the publication can be downloaded, the Ensembl version 93 code gEVAL is built on is not publicly available anymore and this is sadly outside our influence. The 2013 publication pertains to all data provided at https://geval.sanger.ac.uk/.

gEVAL has moved on over nearly a decade and has evolved from a low throughput vehicle for reference curation to a high throughput, fully automated assembly analyser that takes its strength from being totally integrated into the institute's data infrastructure, allowing immediate data retrieval from multiple sources. It is an essential part of the overall assembling pipeline and not promoted as free-standing software. Detangling this to make it publicly available is not possible without additional workforce that we are not funded for. All gEVAL databases we build for the assemblies we are curating are publicly available at vgp-geval.sanger.ac.uk/index.html. This site and its sister site mentioned above are both accessible from geval.org.uk.

The current manuscript does NOT focus on gEVAL as a software packet, but rather on the process of assembly curation and its importance for generating high quality assemblies. We describe what we have successfully applied for our purposes whilst fully disclosing the logic around assembly curation and the tools publicly available to design an assembly curation pipeline that fits the requirements of the respective user.

We have amended the manuscript to hopefully explain this better without taking up too much space and distracting from the core message on curation rather than software:

"gEVAL is tied into our local infrastructure and as such sadly not portable, yet fully publicly accessible at geval.org.uk."

"The pipeline that GRIT deploys has much evolved since its first implementation [10], and is now so closely tied into the Wellcome Sanger Institute's internal data structure that it cannot be ported, but is described here as an example of a successful implementation that mixes automated and manual processes and significantly improves genome assemblies in a time and resource sensitive way that allows its use within high-throughput projects. All assembly projects loaded into gEVAL are publicly accessible at geval.org.uk."

The gEVAL functionality can be largely replicated with any tool that visualises sequence and accepts sequence annotation overlays. In the manuscript, we recommend to use ASSET as it also provides the multi-data analyses that are the core of gEVAL. We have extended the text to make this clearer by adding

"ASSET evaluates multiple data types in parallel and is therefore an excellent tool to assess and visualise potential misassemblies [32]."

3) Finally, I think that it would be hugely beneficial for readers to have a workflow figure with their recommendations incorporated from the initial coherence check to final ordering and orientation.

Thank you for this excellent suggestion, we completely agree and have provided a workflow (Fig. 1) to summarise our recommendations for assembly curation.

Specific comments:

line 100 - extra period at end of sentence

removed

line 106 - spell out Segmental Duplication Assembler.

done

line 113 - comma after "For polishing"

inserted

line 117 - clarify that they can be assembled independently from the raw reads used for genome assembly.

amended: “They can be assembled independently from the raw reads, e.g. using the mitoVGP pipeline”

line 118-119 - This is confusing, it was just stated above that the organelle genome must be included for polishing and now this says to process it independently.

changed from
“Contigs/scaffolds that represent the organelle genomes should be identified and processed independently of the primary, nuclear assembly.”
to
“Contigs/scaffolds that represent the organelle genomes should be identified and submitted as such to the INSDC archives.‟
to specify that the different handling applies to the submission process.

line 208 - typo "gata"

corrected to “data”

line 223 - provide a link to a public code repository with the nextflow pipeline

This pipeline is intricately intertwined with the Sanger infrastructure and it would require additional staff to rewrite it to make it publicly useable. A similar public pipeline already exists, and we have added it to the manuscript:

“Before being loaded into gEVAL, all assemblies are run through a nextflow [39] pipeline that performs contamination detection and separation or removal as described in Table 1, combined with removal of trailing Ns [39]. Brief manual checking of the results prevents the erroneous removal of regions likely derived from horizontal gene transfer. This pipeline was inspired by the contamination checking process conducted by Genbank [40].”

Figure 1: This example is a little confusing. It looks like some of the bionano maps agree with the join and span the drop in pacbio read coverage.

The confusion was likely caused by the lack of annotation on the in silico digest tracks and the BioNano map alignments’ colour scheme. We have extended the feature track annotation to the in silico tracks and added further explanations to the figure legend (yellow = aligned, beige = not aligned BioNano map).

Close
