# Supplementary material for: Significantly improving the quality of genome assemblies through curation
Source: Gigascience. 2021 Jan 9;10(1):giaa153. doi: 10.1093/gigascience/giaa153 (PMC7794651; doi:10.1093/gigascience/giaa153)
Supplement: giaa153_Reviewer_1_Report_Original_Submission — Alan L Archibald -- 9/28/2020 Reviewed [file giaa153_reviewer_1_report_original_submission.pdf]

Reviewer Report

Title: Significantly improving the quality of genome assemblies through curation

Version: Original Submission **Date:** 9/28/2020

Reviewer name: Alan L Archibald

Reviewer Comments to Author:

The authors provide a concise overview of issues that arise during efforts to establish a reference quality genome sequence assembly, especially for organisms with complex genomes. The relevant published literature and software sources are cited. Whilst the authors' own infrastructure for reviewing and correcting genome assemblies is an in-house bespoke system that is not portable they describe the key processes involved in reviewing and assessing genome assemblies. This brief editorial / review provides a useful checklist for groups generating genome assemblies. Whilst the generation of the primary sequence data from which a first pass contig level assembly can be built is readily within the capacity of well-founded and funded research groups, the conversion of the resulting contigs into a high quality chromosome level assembly requires time and skill. This review provides a useful guide to navigating this transition and those who aspire to contribute to the growing resource of high quality reference genomes would be well served by reading this guide.
This guide is largely set in the context of the current widely adopted paradigm of single pseudo-haploid representations of an organism's genome. As some of the errors that the procedures described in this paper seek to address concern the challenges of resolving an individual's different haplotypes some comment on graph based genome approaches to capture rather than 'resolve' such haplotypic differences would be appropriate.

Methods

Are the methods appropriate to the aims of the study, are they well described, and are necessary controls included? Choose an item.

Conclusions

Are the conclusions adequately supported by the data shown? Choose an item.

Reporting Standards

Does the manuscript adhere to the journal's guidelines on minimum standards of reporting? Choose an item.

Choose an item.

Statistics

Are you able to assess all statistics in the manuscript, including the appropriateness of statistical tests used? Choose an item.

Quality of Written English

Please indicate the quality of language in the manuscript: Choose an item.

Declaration of Competing Interests

Please complete a declaration of competing interests, considering the following questions:

- Have you in the past five years received reimbursements, fees, funding, or salary from an organisation that may in any way gain or lose financially from the publication of this manuscript, either now or in the future?
- Do you hold any stocks or shares in an organisation that may in any way gain or lose financially from the publication of this manuscript, either now or in the future?
- Do you hold or are you currently applying for any patents relating to the content of the manuscript?
- Have you received reimbursements, fees, funding, or salary from an organization that holds or has applied for patents relating to the content of the manuscript?
- Do you have any other financial competing interests?
- Do you have any non-financial competing interests in relation to this paper?

If you can answer no to all of the above, write 'I declare that I have no competing interests' below. If your reply is yes to any, please give details below.

I declare that I have no competing interests.

I agree to the open peer review policy of the journal. I understand that my name will be included on my report to the authors and, if the manuscript is accepted for publication, my named report including any attachments I upload will be posted on the website along with the authors' responses. I agree for my report to be made available under an Open Access Creative Commons CC-BY license (http://creativecommons.org/licenses/by/4.0/). I understand that any comments which I do not wish to be included in my named report can be included as confidential comments to the editors, which will not be published.

Choose an item.

To further support our reviewers, we have joined with Publons, where you can gain additional credit to further highlight your hard work (see: https://publons.com/journal/530/gigascience). On publication of this paper, your review will be automatically added to Publons, you can then choose whether or not to claim your Publons credit. I understand this statement.

Yes Choose an item.
