# Supplementary material for: Significantly improving the quality of genome assemblies through curation
Source: Gigascience. 2021 Jan 9;10(1):giaa153. doi: 10.1093/gigascience/giaa153 (PMC7794651; doi:10.1093/gigascience/giaa153)
Supplement: giaa153_Reviewer_2_Report_Original_Submission — Benjamin D Rosen -- 10/7/2020 Reviewed [file giaa153_reviewer_2_report_original_submission.pdf]

Reviewer Report

Title: Significantly improving the quality of genome assemblies through curation

Version: Original Submission **Date:** 10/7/2020

Reviewer name: Benjamin D Rosen

Reviewer Comments to Author:

The authors provide much welcome guidelines and recommendations for genome assembly curation derived from their experience curating hundreds of assemblies. Their recommendations are clear but the manuscript could benefit from more examples of what misassembly signals look like in different technologies. The authors mention that gEVAL is tied into their local infrastructure and not portable, but the original gEVAL manuscript mentions that it is downloadable for use with any organism. It should be made more clear why gEVAL cannot be used. If gEVAL indeed cannot be used outside of their group, it would be nice to see how similar views could be generated with publicly available tools. Finally, I think that it would be hugely beneficial for readers to have a workflow figure with their recommendations incorporated from the initial coherence check to final ordering and orientation.
Specific comments:
line 100 - extra period at end of sentence
line 106 - spell out Segmental Duplication Assembler.
line 113 - comma after "For polishing"
line 117 - clarify that they can be assembled independently from the raw reads used for genome assembly.
line 118-119 - This is confusing, it was just stated above that the organelle genome must be included for polishing and now this says to process it independently.
line 208 - typo "gata"
line 223 - provide a link to a public code repository with the nextflow pipeline
Figure 1: This example is a little confusing. It looks like some of the bionano maps agree with the join and span the drop in pacbio read coverage.

Methods

Are the methods appropriate to the aims of the study, are they well described, and are necessary controls included? Choose an item.

Conclusions

Are the conclusions adequately supported by the data shown? Choose an item.

Reporting Standards

Does the manuscript adhere to the journal's guidelines on [minimum standards of reporting?]() Choose an item.

Choose an item.

Statistics

Are you able to assess all statistics in the manuscript, including the appropriateness of statistical tests used? Choose an item.

Quality of Written English

Please indicate the quality of language in the manuscript: Choose an item.

Declaration of Competing Interests

Please complete a declaration of competing interests, considering the following questions:

- Have you in the past five years received reimbursements, fees, funding, or salary from an organisation that may in any way gain or lose financially from the publication of this manuscript, either now or in the future?
- Do you hold any stocks or shares in an organisation that may in any way gain or lose financially from the publication of this manuscript, either now or in the future?
- Do you hold or are you currently applying for any patents relating to the content of the manuscript?
- Have you received reimbursements, fees, funding, or salary from an organization that holds or has applied for patents relating to the content of the manuscript?
- Do you have any other financial competing interests?
- Do you have any non-financial competing interests in relation to this paper?

If you can answer no to all of the above, write 'I declare that I have no competing interests' below. If your reply is yes to any, please give details below.

I declare that I have no competing interests

I agree to the open peer review policy of the journal. I understand that my name will be included on my report to the authors and, if the manuscript is accepted for publication, my named report including any attachments I upload will be posted on the website along with the authors' responses. I agree for my report to be made available under an Open Access Creative Commons CC-BY license (http://creativecommons.org/licenses/by/4.0/). I understand that any comments which I do not wish to be included in my named report can be included as confidential comments to the editors, which will not be published.

Choose an item.

To further support our reviewers, we have joined with Publons, where you can gain additional credit to further highlight your hard work (see: https://publons.com/journal/530/gigascience). On publication of this paper, your review will be automatically added to Publons, you can then choose whether or not to claim your Publons credit. I understand this statement.

Yes Choose an item.
